# In Vitro Evaluation of Bis-3-Chloropiperidines as RNA Modulators Targeting TAR and TAR-Protein Interaction

**DOI:** 10.3390/ijms23020582

**Published:** 2022-01-06

**Authors:** Alice Sosic, Giulia Olivato, Caterina Carraro, Richard Göttlich, Dan Fabris, Barbara Gatto

**Affiliations:** 1 Department of Pharmaceutical and Pharmacological Sciences, University of Padova, Via Francesco Marzolo 5, 35131 Padova, Italy; alice.sosic@unipd.it (A.S.); giulia.olivato94@gmail.com (G.O.); caterina.carraro.2@phd.unipd.it (C.C.); 2 Institute of Organic Chemistry, Justus Liebig University Giessen, Heinrich-Buff-Ring 17, 35392 Giessen, Germany; Richard.Goettlich@org.chemie.uni-giess; 3 Department of Chemistry, University of Connecticut, 55 North Eagleville Rd., Storrs, CT 06269, USA; dan.fabris@uconn.edu

**Keywords:** RNA targeting, RNA-based interactions, bis-3-chloropiperidines

## Abstract

After a long limbo, RNA has gained its credibility as a druggable target, fully earning its deserved role in the next generation of pharmaceutical R&D. We have recently probed the trans-activation response (TAR) element, an RNA stem–bulge–loop domain of the HIV-1 genome with bis-3-chloropiperidines (B-CePs), and revealed the compounds unique behavior in stabilizing TAR structure, thus impairing in vitro the chaperone activity of the HIV-1 nucleocapsid (NC) protein. Seeking to elucidate the determinants of B-CePs inhibition, we have further characterized here their effects on the target TAR and its NC recognition, while developing quantitative analytical approaches for the study of multicomponent RNA-based interactions.

## 1. Introduction

**Figure 1:**
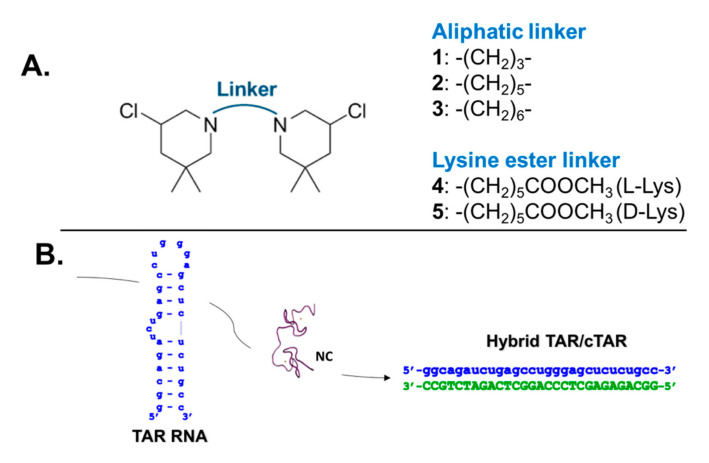
(**A**) Chemical structure of bis-3-chloropiperidines analyzed in this work. (**B**) Sequence and secondary structure of construct replicating TAR RNA which was employed in our assays. NC chaperone activities on TAR RNA led to the formation of the hybrid TAR/cTAR.

The last few years have witnessed a remarkable surge of interest in RNA as a putative therapeutic and research tool, which has led to a greater understanding of structure/function relationships and the discovery of fundamental roles in infection, inflammation, and other disease conditions [1,2]. Likewise, renewed attention has been devoted to studying the roles of RNA-protein interactions in the aforementioned conditions. As a consequence, the hunt for drug-like ligands able to modulate RNA functions, as well as RNA-protein interactions, has culminated in the approval of the first small molecule in a new generation of RNA-targeting drugs [3], bringing this biopolymer to the forefront as an innovative druggable target [1,4,5,6,7]. At the same time, it has become clear that progress in the identification of new inhibitors is severely limited by the dearth of technologies and biophysical tools for the analysis and characterization of the interactions established by RNA, which still lag far behind those available for other types of biopolymers, most notably proteins. In this context and to address these needs, we have demonstrated that the concerted application of electrophoretic and mass spectrometry (MS)-based techniques is capable of supporting the characterization of multiple covalent modifications introduced onto RNA by small molecules such as bis-3-chloropiperidines (B-CePs) [8,9]. B-CePs are piperidine- based alkylating agents characterized by a sterically restricted reactive moiety which readily attacks nucleophiles in nucleic acids causing adduct formation [10,11,12,13]. Nucleic acids alkylation by B-CePs was extensively examined, confirming B-CePs formation of an aziridinium ion by intramolecular nucleophilic substitution reaction. The extremely unstable species thus formed undergoes nucleophilic attack most often by N-7 of guanines, forming a covalent bond with the nucleic acid and resulting in both mono-functional and bi-functional adducts [8,9,10]. The chemistry of linkers connecting the two reactive centers of B-CeP scaffolds shown in Figure 1A was thoroughly investigated as a synthetic tool to tailor the reactivity of these compounds. Analyzing reactions with water and DNA, we found that flexible linkers as in **1**–**3** enhanced B-CePs reactivity [12], while the ester functionality as in the lysine ester linkers **4** and **5** reduced the reactivity compared to the linear alkyl analogs [11]. The comprehensive characterization of reaction products obtained by compound **1** with selected RNA constructs revealed the formation of distinctive inter-molecular cross-links between guanines in double stranded sequences [8,9], which had not been observed for any DNA construct previously examined [10,11,12,13]. We leveraged this unique feature to verify the location of guanines involved in long-range quaternary interactions in highly structured RNA systems, such as the kissing loop (KL) complex formed by two copies of the dimerization initiation site (DIS) of the HIV-1 genome [8]. We further demonstrated the B-CePs’ ability to cross-link base-paired regions within TAR RNA [9], a stem-loop structure of great pharmacological relevance for the development of antiviral drugs. Follow-up studies aimed at elucidating the determinants of B-CePs activity towards RNA indicated that compound [1,2,3], which are characterized by progressively longer alkyl linkers between reactive moieties (Figure 1A), were capable of stabilizing TAR secondary structure, as well as affecting the functions of TAR-processing proteins in vitro [9]. The fact that such effects were again less prominent for the lysine ester compounds **4** and **5** (Figure 1A) implied that linker size and composition could be determinant also in the formation of pertinent TAR-protein complexes. 

We tested this hypothesis while elucidating B-CePs inhibitory activities on specific RNA–protein interactions. We investigated the effects of the compounds shown in Figure 1A on the specific interactions established by TAR RNA with the HIV-1 nucleocapsid (NC) protein, a highly conserved nucleic acids chaperone involved in many essential steps of the viral lifecycle [14,15]. Indeed, NC is known to mediate reverse transcription by destabilizing TAR’s secondary structure, enabling annealing to the reverse-transcribed complementary cTAR DNA, and promoting the formation of a stable TAR/cTAR heteroduplex (Figure 1B) [14,16,17]. The observation that TAR alkylation by the B-CePs in Figure 1A impaired the structure-destabilizing activity of NC [9], prompted a systematic evaluation of their effects onto TAR-NC binding to better understand their putative inhibitory properties. This evaluation involved accomplishing: (i) the qualitative and quantitative analysis of B-CePs-TAR adducts for the selected test compounds; (ii) the quantification of the crosslinks induced within the TAR RNA hairpin structure; (iii) the quantification of B-CePs inhibition of TAR-NC binding; and (iv) analogous analyses on samples in which TAR RNA was replaced by its complementary cTAR DNA construct (Figure 1B). The results afforded new valuable insights into the mechanisms of B-CePs at the molecular level. At the same time, the experiments provided the opportunity to assess the merits of our concerted analytical strategy for the in vitro investigation of multicomponent complexes involving RNA. 

## 2. Results and Discussion

### 2.1. Aliphatic Linker Improves B-CePs Reactivity towards TAR

**Figure 2:**
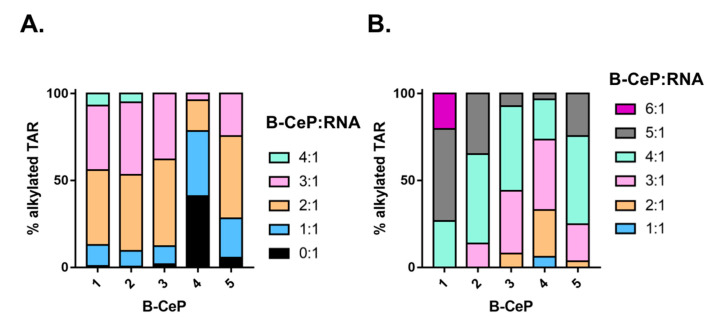
Histograms comparing the percentage of alkylated TAR RNA substrate showing the relative abundances of the detected adducts stoichiometries for B-CePs **1**–**5**. Adduct/TAR ratios are indicated by different colors in the legends. Data were obtained from analyses of the ESI-MS spectra of reaction mixtures containing TAR RNA (1 μM) and either 10 μM (**A**) or 50 μM (**B**) of each B-CePs, incubated for 2 h at 37 °C (see Section 3 for details).

To elucidate the effects of the different linkers connecting the reactive moieties of B-CePs, we estimated the relative abundance of the different alkylated RNA species obtained upon reactions with the test compounds. Briefly, spectra acquired from samples obtained by reacting 10 μM of compounds **1**–**5** with 1 μM TAR for 2 h at 37 °C were utilized to quantify the relative percentage of each observed product (see Section 3 for details). The cumulative percentages of alkylation products were plotted in histogram form and reported in Figure 2A. The results clearly showed that all compounds were capable of converting most of the initial substrate into reaction products under the selected conditions, but significant differences were immediately evident, which could be ascribed to the type and length of linker structure. For instance, B-CePs **1**–**3** bearing progressively longer aliphatic linkers produced greater abundances of higher stoichiometry adducts. In contrast, the lysine-ester derivatives **4** and **5** displayed larger fractions of unreacted RNA and lower adduct stoichiometries. Interestingly, the D configuration of the Lys linker of **5** seems to improve its reactivity towards RNA compared to the natural L-Lys analogue **4**. **1** and **2** exhibited the greater reactivities in the series with stoichiometries up to 4:1, whereas **3** exhibited only up to 3:1 adducts. Increasing the compound concentrations 5-fold to 50 μM produced analogous reactivity trends under otherwise identical reaction conditions (Figure 2B). In this case, however, the increased concentrations resulted in the complete conversion of the initial substrate regardless of reactivity. With the exception of **3**, the aliphatic B-CePs resulted to be again more reactive than the Lys-derivatives, with compound **1** displaying up to 6:1 B-CeP:RNA ratio. The distribution of the abundances of the various alkylation products suggested a putative scale of reactivity towards TAR, in which aliphatic and Lys-compounds were at the opposite ends: **1** resulted to be the most reactive, followed by **2** and **3**, whereas **4** was less reactive than **5** (**1** > **2** > **3** > **5** > **4**). These results are in good agreement with the previous observation that the reactivity of Lys-derivatives is lower compared to the aliphatic B-CePs [18].

### 2.2. B-CePs Adducts Inhibit NC Binding to TAR

**Figure 3:**
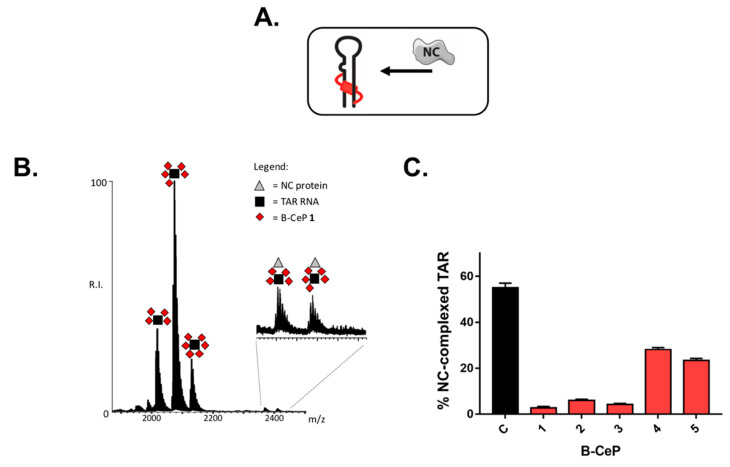
(**A**) Schematic representation of the experimental procedure employed to evaluate the inhibition of NC binding to TAR. (**B**) Representative ESI-MS spectrum of samples obtained by adding NC protein to TAR pre-treated with B-CeP **1** (50:1 compound:RNA ratio) for 2 h at 37 °C. NC protein and TAR are 1 μM each. The spectra were recorded in 150 mM ammonium acetate. The inset shows an enlarged view of the detected ternary complexes. (**C**) Histogram comparing the percentage of NC-complexed TAR in the absence and in the presence of alkylation adducts produced by B-CePs **1**–**5**.

The ability of selected B-CePs to covalently modify TAR, decorating its structure with stable mono- and bi-functional adducts, raised the possibility that such alkylation might affect NC binding to its TAR RNA substrate to an extent correlated with the length and type of linker moiety. To test this hypothesis, we evaluated samples in which TAR was pre-reacted with each B-CeP and then incubated with NC protein to promote binding. The outcome of each experiment was determined by electrospray ionization-mass spectrometry (ESI-MS) under native conditions, which has been shown capable of enabling the detection of non-covalent complexes between RNA and NC [19,20,21,22]. Control experiments were performed by incubating equimolar amounts of full-length NC protein with TAR RNA construct (i.e., 1 μM concentration of each, see Section 3) in the absence of compound, which confirmed the formation of stable 1:1 TAR∙NC complexes (Figure S1). In subsequent experiments, the same equimolar concentration of NC was added to 1 μM samples of TAR RNA, which had been pre-reacted separately with 50 μM of compounds **1**–**5** as described above (Figure 3A). The representative spectrum in Figure 3B, which was obtained after brief incubation to enable the establishment of any possible binding equilibrium, revealed that NC retained the ability to bind with 1:1 stoichiometry the alkylated TAR products, but not nearly with the same affinity exhibited by the unmodified construct in the absence of reaction. Analogous results were observed for all the compounds tested in the study.

The signal intensities of the various species were employed with proper precautions to estimate the percentages of free and bound components in solution, as detailed in Section 3. For example, the histogram in Figure 3C reports the overall percentages of bound NC detected in complexes with either unmodified TAR in the control sample, or the B-CePs-RNA adducts produced by alkylation. The values confirmed that alkylation had significantly reduced the strength of protein binding in a compound-specific manner. More specifically, the trend followed very closely the relative scale of reactivity toward the RNA substrate, thus suggesting a possible correlation between adduct stoichiometry and binding inhibition. This possibility was supported by the observation of greater percentages of bound NC for the Lys-linker compounds **4** and **5**, which had afforded lower adduct stoichiometries. Conversely, a lower incidence of protein binding was observed for the aliphatic-linker compounds **1**–**3**, which had produced higher adduct distributions. In particular, compound **1**—which had produced the greatest number of adducts (i.e., 6:1)—was also the B-CeP that exhibited by far the most pronounced inhibition of NC binding. Nevertheless, not only the B-CePs-induced stoichiometry of TAR alkylation but also the quality of the linker impairs the binding of NC to the alkylated TAR. 

### 2.3. Mapping the Aliphatic B-CePs Crosslinks on TAR

**Figure 4:**
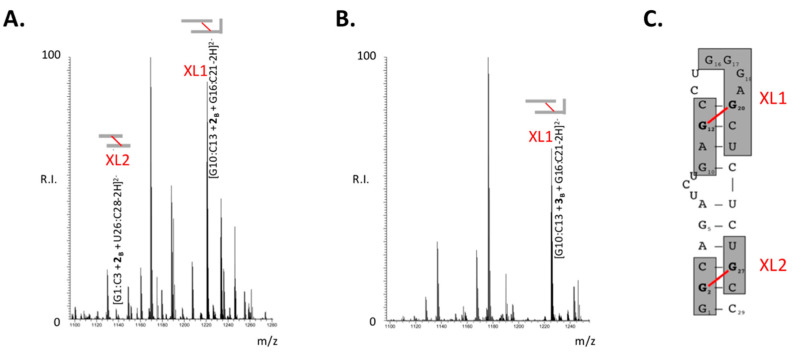
Representative ESI-MS data obtained from TAR RNA (1 μM) samples reacted with 10 μM of either B-CeP **2** (**A**) or B-CeP **3** (**B**) followed by digestion with RNAse A (see Section 3 for details). For the sake of clarity, only the relevant crosslinking products are labeled in each spectrum. Numerous other species corresponding to hydrolytic products were identified (see Figure S2). Cleavage products are identified by the first and last base (e.g., G10:C13) and the number of the respective bi-functional alkylator (i.e., **2** or **3**). (**C**). Cartoon of the B-CePs-induced bi-functional alkylation products named XL1 and XL2 within the TAR RNA secondary structure.

The observed correlation between alkylation stoichiometry and binding properties could be explained by putative structural effects induced by chemical modification, which might result in occupancy or distortion of the protein binding site(s) present on the TAR construct. To gain additional insights into the inhibition mechanism, we sought to map the position of B-CePs adducts onto the RNA structure. In previous studies, we demonstrated that these compounds possessed the exquisite ability to form bifunctional cross-links that bridged across opposing RNA strands [8]. We demonstrated also that the crosslinks produced by B-CeP **1** across key guanines in the TAR hairpin prevented both thermal and NC-mediated melting of the double-stranded stem [9]. We have now investigated the effects of lengthening the aliphatic linker of the B-CePs series **1**–**3** by comparing the positions of the nucleotides involved in the bridging conjugates. This task was accomplished by performing RNAse A digestion of the adducts followed by mass-spectrometric characterization of the cleavage products. The representative data obtained from TAR RNA treated with either compound **2** or **3** (Figure 4A,B, respectively) shared many similarities consistent with the underlying TAR structure, including cross-linked products obtained from the same region of the hairpin, which had been previously proven susceptible to treatment with **1** [9]. Product labeled XL1 (XL, cross-link) corresponded to TAR fragments G10:C13 and G16:C21 bridged by either **2** or **3** into G10:C13 + **2_B_** + G16:C21 and G10:C13 + **3_B_** + G16:C21, respectively. The compound **2** products included an additional conjugate present also in compound **1** samples but absent in those of compound **3**, which corresponded to G1:C3 bridged to U26:C28 into the cross-linked species G1:C3 + **2_B_** + U26:C28 (labeled XL2 in Figure 4A). The positions of the XL1 and XL2 cross-linked species onto the TAR structure are visualized as shaded sequences in Figure 4C, and their features are summarized in Table S1.

Signal intensities were once again employed to estimate the percentage of conjugated XL1 and XL2 species observed in the various spectra (see Section 3), with the aim to infer valuable information on the ability of the aliphatic B-CePs to produce these types of cross-links. The results were summarized in Table 1, which rated the compounds as excellent (✓✓), limited (✓), or negligible (✗) cross-linkers in the context of the TAR RNA structure. At the low concentrations tested here, these B-CePs were all capable of producing comparable percentages of the cross-linked species XL1. In contrast, their ability to produce XL2 decreased progressively in the **1**–**3** series as a function of linker length, in a remarkable example of structure-activity relationship (SAR). It should be noted that, consistent with our previous observations [9], the cartoon in Figure 4C assigned the XL1 cross-link as bridging G12 to G20. However, the progressively longer aliphatic linkers of B-CePs **2** and **3** may place G12 within striking distance of other susceptible guanines in the G-rich loop (i.e., G16, G17, and G18). The ability to form alternative conjugates could be supported also by the intrinsic flexibility of the loop structure and by the rather unprotected, accessible context of these single-stranded nucleotides. On the other hand, the G2 and G27 nucleotides involved in XL2 are located in a base-paired stretch that narrowly constrains their mutual distance, as well as the distances between them and other possible guanines in the region (Figure 4C). The structural context is such that only compound **1** may be capable of optimally bridging G2 to G27, whereas compound **2** may be less permissive. The length of the –(CH_2_)_6_– linker characteristic of compound **3** may just exceed the distance between such nucleotides without reaching those necessary to bridge alternative targets. 

### 2.4. B-CePs Alkylation Affects NC-Mediated TAR Interactions with cTAR

**Figure 5:**
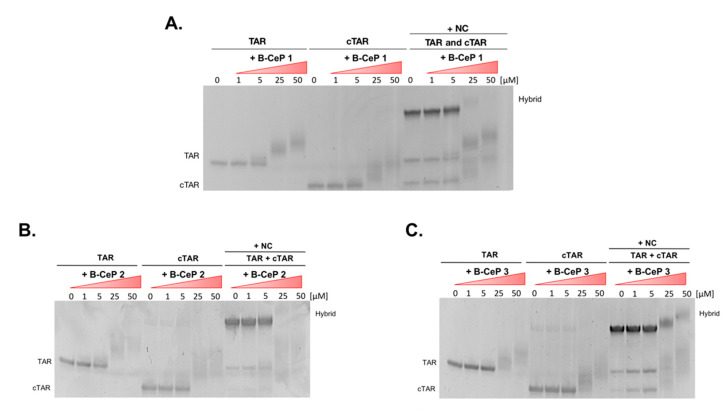
Non-denaturing polyacrylamide gel electrophoresis (PAGE) showing the concentration-dependence of the interaction of (**A**) B-CeP **1**, (**B**) B-CeP **2**, (**C**) B-CeP **3**, with TAR and cTAR, and the relative effects on the NC-mediated formation of the TAR/cTAR hybrid. Folded TAR and cTAR hairpin 1 μM were incubated with increasing concentrations of compound (0, 1, 5, 25, 50 μM) for 2 h at 37 °C in BPE buffer in the absence of the protein. Identical TAR and cTAR samples, alkylated upon reaction with B-CePs, were mixed together and incubated with 8 μM NC protein for 15 min at room temperature.

If the binding experiments exposed the negative impact of B-CePs modifications on TAR-NC interactions, they also hinted to the possibility that similar effects might influence the specific interactions with the cognate cTAR structure, which are essential to reverse transcription process [17]. It has been shown that transient melting of the TAR and cTAR structures mediated by NC is an essential step in promoting the formation of their stable heteroduplex [16]. The presence of inter-strand cross-links produced by B-CePs may prevent the melting of the double-stranded stems, thus indirectly inhibiting the structural rearrangements promoted by the NC’s chaperone activities. This hypothesis, however, would require that cTAR be affected by B-CePs in similar fashion. We therefore proceeded to compare the outcomes of B-CePs **1**–**3** alkylation of both TAR and cTAR, and the effects of such alkylation reactions on the NC-mediated formation of the TAR/cTAR hybrid. For this purpose, we employed electrophoretic mobility shift assay (EMSA), a simple electrophoretic approach capable of detecting the formation of stable nucleic acids adducts by retardation of electrophoretic migration [9], and of resolving between the nucleic acid monomers and the hybrid [22]. The three aliphatic B-CePs were reacted with separate TAR and cTAR under identical conditions in the absence of the NC protein (see Section 3) and directly loaded in the gel system. In parallel, identical samples were prepared and, after B-CePs reactions, alkylated TAR and cTAR were mixed and incubated with the NC protein (see Section 3) to promote the formation of the TAR/cTAR hybrid. All the results were reported in Figure 5. The results clearly demonstrated the ability of NC to mediate the formation of annealed TAR/cTAR heteroduplex in the absence of compounds. In contrast, inhibition of the chaperone activity was evidenced by the dose-dependent decrease of annealed heteroduplex in the samples obtained after reaction of the nucleic acid constructs with B-CePs **1**–**3** (right part of each gel system in Figure 5). The decreased hybrid formation is mirrored by the detection of its individual cTAR and TAR components, which appear clearly modified by B-CePs, suggesting that NC-mediated interactions of TAR with cTAR is actually impaired by the B-CePs alkylation. In fact, the data clearly indicated that all compounds were capable of reacting with both individual constructs in the absence of NC, as evidenced by the upward shift of TAR and cTAR bands (left and middle parts of each gel system in Figure 5). In the case of cTAR; however, the bands observed at the higher compound concentrations were not nearly as resolved as those observed for TAR. Considering that ‘smearing’ lanes are characteristic of complex sample mixtures, these results suggested that cTAR supported the formation of adducts with greater structural diversity than those supported by its RNA counterpart.

**Figure 6:**
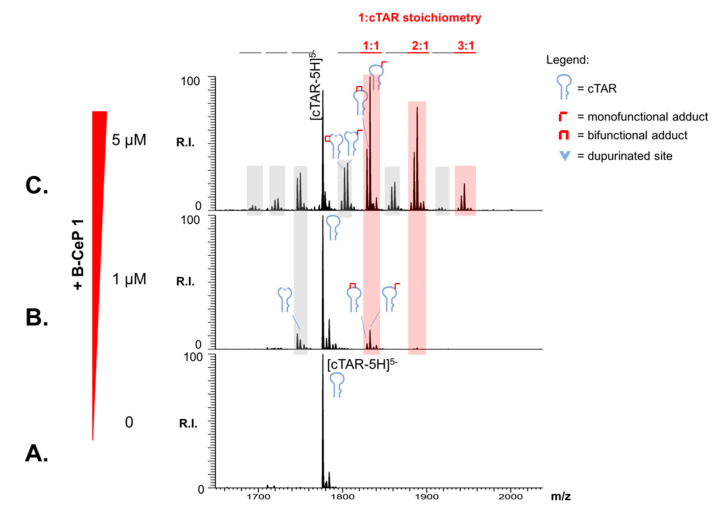
Representative ESI-MS spectra of reaction mixtures obtained by incubating cTAR (1 μM) with increasing B-CeP **1** ((**A**):0, (**B**):1, (**C**):5 μM) at 37 °C for 2 h in BPE buffer (see Section 3). The initial unmodified substrate was immediately identified with a mass of 8884.5 u matching the value calculated from the DNA sequence. Multiple stoichiometries corresponding to combinations of mono- and bi-functional adducts were also detected, as indicated in red. Depurination products due to hydrolysis of alkylated guanines were identified and highlighted in gray. Lower intensity signals near free/adducted species consist of typical sodium and ammonium adducts. Only the region containing the 5-charge state is shown for the sake of clarity.

The products of B-CeP reaction with cTAR DNA were analyzed also by ESI-MS to elucidate the molecular details. Figure 6 shows representative data obtained by mixing 1 μM cTAR with increasing concentrations of compound **1** (0, 1, and 5 μM) and allowing them to react for 2 h at 37 °C. The spectra contained intense signals corresponding to unreacted cTAR, as well as several **1** adducts. Such products were readily assigned to combinations of mono- and bi-functional adducts with stoichiometries increasing from 2:1 to 3:1 in a concentration-dependent manner (shaded in red in Figure 6B,C). Multiple products with masses lower than the corresponding adducts (gray highlights in Figure 6B,C) were also observed, which were attributed to the loss of alkylated guanines caused by hydrolysis of the corresponding adduct [10]. The depurination process, which was observed also in EMSA data (Figure 5), is consistent with the weakening of the N-glycosidic bond associated with the alkylation of the N-7 position of the fused rings system. These results confirmed that B-CePs-induced depurination, which was previously observed for single and double-stranded DNA model systems [10], can take place also on a DNA structure of biological relevance as a substrate for viral protein binding. It should be noted that, at the low compound concentration and short incubation time employed here, no trace could be detected of the strand-cleavage activity reported for this class of compounds with model DNA strands [10]. The absence of such activity and the fact that compound **1** elicited the lowest cytotoxicity in the aliphatic series **1**–**3** [18] could raise some interest in this analog as an antiviral lead compound. The formation of alkylation also on this DNA stem-loop supports the proposed mechanism by which B-CeP modifications on both TAR and cTAR constructs inhibit the formation of the TAR/cTAR hybrid mediated by NC. 

## 3. Materials and Methods

### 3.1. Nucleic Acid Substrates and Protein

All oligonucleotides were synthesized by Metabion International AG (Martinsried, Germany) and stored at −20 °C in 10 mM Tris-HCl pH 8.0. TAR is the 29-mer RNA sequence 5′-GGCAGAUCUGAGCCUGGGAGCUCUCUGCC-3′ and cTAR is its DNA complementary sequence 5′-GGCAGAGAGCTCCCAGGCTCAGATCTGCC-3′. Dilutions were made in DEPC-treated water (Thermo Fisher Scientific, Monza, Italy). The typical folding procedure consisted of snap-cooling: either TAR RNA or cTAR DNA diluted in 1X BPE buffer (NaH_2_PO_4_ 0.2 mM, Na_2_HPO_4_ 0.6 mM, Na_2_EDTA 0.1 mM, pH 7.4) was heated to 95 °C for 5 min and then ice-cooled in order to assume the proper hairpin structure. The full-length recombinant NC protein was obtained in house as reported [23]. 

### 3.2. Chemical Reagents

Bis-3-chloropiperidines **1**–**5** (Figure 1A) were synthesized in house, as previously described [11,12]. Aliquots of chemical probes were freshly prepared by diluting a 10 mM DMSO stock in MilliQ water and were instantly reacted with the nucleic acid substrate to avoid the typical quenching effects of the aqueous environment. All the other chemical reagents, including salts and solvents, were purchased from Sigma-Aldrich (Milan, Italy).

### 3.3. Probing Reactions

Based on the final aim of the experiment, different reaction conditions were explored, including different substrates and different compound to substrate ratios. Incubation time is instead consistent in all the experiments: nucleic acid solutions had B-CePs **1**–**5** added to them and then were incubated at 37 °C for 2 h. Case by case reaction conditions are detailed within the text. Typical reaction mixtures consisted of either TAR RNA or cTAR DNA, properly folded in 1X BPE buffer as described above.

### 3.4. Mass Spectrometric Analysis

Samples prepared in BPE were buffer-exchanged by performing ethanol precipitation in the presence of 1 M of ammonium acetate. In case of reaction mixtures, the treatment also served to achieve reaction quenching. Samples were re-dissolved and diluted in 150 mM ammonium acetate (pH adjusted to 7.0) to achieve a final 1 μM concentration of total nucleic acid substrate. Samples were analyzed by direct infusion electrospray ionization (ESI) on a Thermo Fisher Scientific (West Palm Beach, CA, USA) LTQ-Orbitrap Velos mass spectrometer. The analyses were performed in nanoflow mode by using quartz emitters produced in-house by using a Sutter Instruments Co. (Novato, CA, USA) P2000 laser pipette puller. Up to 5 μL samples were typically loaded onto each emitter by using a gel-loader pipette tip. A stainless-steel wire was inserted in the back-end of the emitter to supply an ionizing voltage that ranged between 0.8 and 1.2 kV. The source temperature and desolvation conditions were adjusted by closely monitoring the incidence of ammonium adducts and water clusters. Data were processed by using Xcalibur 2.1 software (Thermo Scientific, West Palm Beach, CA, USA).

### 3.5. Inhibition of NC Binding to TAR RNA

Possible effects induced by B-CePs on the specific binding of NC protein to TAR substrate were evaluated by analyzing samples in which TAR was pre-reacted with each B-CeP (50:1 compound:RNA ratio) for 2h at 37 °C and then incubated with equimolar amount of NC protein (1 μM each) in 150 mM ammonium acetate. ESI-MS performed under nondenaturing conditions was applied to unambiguously identify all species present at equilibrium in solution. Samples were analyzed in negative ion mode via direct infusion nanospray ionization as described above on a Synapt G2 HDMS traveling-wave ion mobility spectrometry (IMS) mass spectrometer (Waters, Manchester, UK). Data were processed by using Mass Lynx (v 4.1, SCN781, Waters, Manchester, UK) software. To evaluate the binding of the full-length NC to TAR construct, abundances of protein-free and NC-complexed TAR species in each experiment were calculated, expressed as percentage, and compared.

### 3.6. Enzymatic Digestion of TAR RNA

Consistently with previous analysis [9], typical reactions consist of a final 1 μM solution of properly folded TAR RNA in 1X BPE buffer with either B-CeP **2** or **3** at final concentrations of 10 μM, i.e., a 10:1 compound/substrate ratio. Reaction mixtures were incubated at 37 °C for 2 h and quenched by ethanol precipitation. Aliquots of unreacted and reacted TAR were submitted to digestion with ribonuclease A (RNAse A) in 150 mM ammonium acetate for 1 h at 37 °C. Samples were stored at -20 °C until immediate analysis by ESI-MS on the Thermo Fisher Scientific (West Palm Beach, CA, USA) LTQ-Orbitrap Velos mass spectrometer, as reported above. Signal intensities of fragments G1:C3 and G10:C13 were used to calculate the abundances of crosslinked species in each experiment and rate the compounds as excellent (XL > 80%), limited (2% < XL < 80%), or negligible (XL < 2%).

### 3.7. Gel Electrophoresis Analysis

Electrophoretic mobility shift assay (EMSA) was used to assess the ability of com- pound **1**–**3** to directly interact with TAR RNA and cTAR DNA constructs, and the effects of such alkylation on the NC-mediated formation of the TAR/cTAR hybrid. Prior to incubation with compound, nucleic acids were heated to 95 °C for 5 min and then ice-cooled in order to assume the proper hairpin structure. TAR and cTAR construct (1 μM) were then incubated with increasing concentrations (from 0 to 50 μM) of B-CePs, at 37 °C for 2 h in 1X BPE buffer. In addition, aliquots of each construct, after reaction with B-CePs as described above, were mixed together, NC solution was added (8 μM), and then the mixture was incubated for other 15 min at room temperature. The samples were then added gel loading buffer containing SDS (GLB_SDS_: Tris-HCl 100 mM, EDTA 4 mM, 50% w/v glycerol, 2% w/v SDS, 0.05% w/v bromophenol blue), at which point they were kept on ice, and resolved by 12% non-denaturing polyacrylamide (PAA) gels containing 1X TBE (Tris Borate EDTA) buffer at room temperature. Unreacted and reacted RNA and DNA on the gel were stained with SybrGreen II® (Invitrogen, Carlsbad, CA, USA). Fluorescence in gel system was detected on a Geliance 600 Imaging System (PerkinElmer, Waltham, MA, USA).

## 4. Conclusions

With the goal of developing RNA-targeting agents, we evaluated B-CePs as RNA cross-linking agents and assessed their ability to interfere with NC-mediated remodeling of TAR and cTAR secondary structures. We determined that B-CeP reaction with TAR RNA produced a series of different effects, in addition to the strong stabilization induced on TAR construct [9]. The aliphatic B-CePs reacted with this substrate more efficiently than the Lys-derivatives. The ensuing products were less conducive to binding by the NC protein, perhaps due to modification of pertinent functional groups involved in the interaction, or to conformational changes in the viral RNA structure. SAR analysis of the aliphatic B-CePs **1**–**3** highlighted the importance of the distance between reactive moieties in driving the formation of covalent bridges between residues placed in different positions of the RNA structure. Cross-linker spacing could thus represent a convenient parameter for finely tuning the reactivity of potential B-CeP-based therapeutics against specific viral structures instead of undesired host targets. Furthermore, it has been shown that putative reactions taking place in the cytoplasmic environment can significantly reduce the extent of damage of genomic DNA caused by the aliphatic compounds **1**–**3** as compared to that of their Lys-analogs **4** and **5** [18]. Taken together, all these considerations make these compounds into suitable candidates for further development as specific RNA-targeting agents for old and new viruses. 

Finally, the results afforded by the TAR/cTAR/NC system demonstrated the merits of the experimental approaches employed here, which could represent a broad base, effective platform for the characterization of multicomponent RNA-based complexes and for the investigation of the effects of small-molecule ligands on their specific interactions. The ability of B-CePs to conjugate nucleotides placed in well-defined structural contexts, and the possibility to adjust their reach by modulating the linker length could pave the way for their utilization as nucleic-acid specific structural probes or interactomic reagents, alongside other bifunctional cross-linkers employed exclusively with protein substrates.

## Figures and Tables

**Table 1:** Evaluation of cross-linking efficiency of the aliphatic B-CePs series **1**–**3**. The production of crosslinked species XL1 and XL2 was assessed by MS analysis and quantified as detailed in Material and Methods section. The observed performance was rated as excellent (✓✓), limited (✓) or negligible (✗).

B-CePs	Linker	XL1	XL2
**1**	-(CH_2_)_3_-	✓✓	✓✓
**2**	-(CH_2_)_5_-	✓✓	✓
**3**	-(CH_2_)_6_-	✓✓	✗

## Data Availability

Not applicable.

## Supplementary Materials

The following are available online at https://www.mdpi.com/article/10.3390/ijms23020582/s1.

## References

[1] Haniff H.S., Knerr L., Chen J.L., Disney M.D., Lightfoot H.L. (2020). Target-Directed Approaches for Screening Small Molecules against RNA Targets. SLAS Discov. Adv. Life Sci. R&D.

[2] Meyer S.M., Williams C.C., Akahori Y., Tanaka T., Aikawa H., Tong Y., Childs-Disney J.L., Disney M.D. (2020). Small molecule recognition of disease-relevant RNA structures. Chem. Soc. Rev..

[3] Sheridan C. (2021). First small-molecule drug targeting RNA gains momentum. Nat. Biotechnol..

[4] Costales M.G., Childs-Disney J.L., Haniff H., Disney M.D. (2020). How We Think about Targeting RNA with Small Molecules. J. Med. Chem..

[5] Disney M.D. (2019). Targeting RNA with Small Molecules to Capture Opportunities at the Intersection of Chemistry, Biology, and Medicine. J. Am. Chem. Soc..

[6] Donlic A., Hargrove A.E. (2018). Targeting RNA in mammalian systems with small molecules. Wiley Interdiscip. Rev. RNA.

[7] Hargrove A.E. (2020). Small molecule–RNA targeting: Starting with the fundamentals. Chem. Comm..

[8] Sosic A., Gottlich R., Fabris D., Gatto B. (2021). B-CePs as cross-linking probes for the investigation of RNA higher-order structure. Nucleic Acids Res..

[9] Sosic A., Olivato G., Carraro C., Göttlich R., Fabris D., Gatto B. (2021). Bis-3-Chloropiperidines Targeting TAR RNA as A Novel Strategy to Impair the HIV-1 Nucleocapsid Protein. Molecules.

[10] Sosic A., Zuravka I., Schmitt N., Miola A., Göttlich R., Fabris D., Gatto B. (2017). Direct and Topoisomerase II Mediated DNA Damage by Bis-3-chloropiperidines: The Importance of Being an Earnest G. ChemMedChem.

[11] Zuravka I., Roesmann R., Sosic A., Göttlich R., Gatto B. (2015). Bis-3-chloropiperidines containing bridging lysine linkers: Influence of side chain structure on DNA alkylating activity. Bioorg. Med. Chem..

[12] Zuravka I., Roesmann R., Sosic A., Wende W., Pingoud A., Gatto B., Göttlich R. (2014). Synthesis and DNA Cleavage Activity of Bis-3-chloropiperidines as Alkylating Agents. ChemMedChem.

[13] Zuravka I., Sosic A., Gatto B., Göttlich R. (2015). Synthesis and evaluation of a bis-3-chloropiperidine derivative incorporating an anthraquinone pharmacophore. Bioorg. Med. Chem. Lett..

[14] Belfetmi A., Zargarian L., Tisné C., Sleiman D., Morellet N., Lescop E., Maskri O., René B., Mély Y., Fossé P. (2016). Insights into the mechanisms of RNA secondary structure destabilization by the HIV-1 nucleocapsid protein. RNA.

[15] Kanevsky I., Chaminade F., Ficheux D., Moumen A., Gorelick R., Negroni M., Darlix J.-L., Fossé P. (2005). Specific Interactions Between HIV-1 Nucleocapsid Protein and the TAR Element. J. Mol. Biol..

[16] Bernacchi S., Stoylov S., Piémont E., Ficheux D., Roques B.P., Darlix J.L., Mély Y. (2002). HIV-1 nucleocapsid protein activates transient melting of least stable parts of the secondary structure of TAR and its complementary sequence. J. Mol. Biol..

[17] Kanevsky I., Chaminade F., Chen Y., Godet J., René B., Darlix J.-L., Mély Y., Mauffret O., Fossé P. (2011). Structural determinants of TAR RNA-DNA annealing in the absence and presence of HIV-1 nucleocapsid protein. Nucleic Acids Res..

[18] Carraro C., Helbing T., Francke A., Zuravka I., Sosic A., De Franco M., Gandin V., Gatto B., Göttlich D.R. (2021). Appended Aromatic Moieties in Flexible Bis-3-chloropiperidines Confer Tropism against Pancreatic Cancer Cells. ChemMedChem.

[19] Turner K.B., Hagan N.A., Fabris D. (2007). Understanding the Isomerization of the HIV-1 Dimerization Initiation Domain by the Nucleocapsid Protein. J. Mol. Biol..

[20] Turner K.B., Kohlway A.S., Hagan N.A., Fabris D. (2009). Noncovalent probes for the investigation of structure and dynamics of protein-nucleic acid assemblies: The case of NC-mediated dimerization of genomic RNA in HIV-1. Biopolym. Orig. Res. Biomol..

[21] Hagan N.A., Fabris D. (2007). Dissecting the Protein–RNA and RNA–RNA Interactions in the Nucleocapsid-mediated Dimerization and Isomerization of HIV-1 Stemloop 1. J. Mol. Biol..

[22] Sosic A., Saccone I., Carraro C., Kenderdine T., Gamba E., Caliendo G., Corvino A., Di Vaio P., Fiorino F., Magli E. (2018). Non-Natural Linker Configuration in 2,6-Dipeptidyl-Anthraquinones Enhances the Inhibition of TAR RNA Binding/Annealing Activities by HIV-1 NC and Tat Proteins. Bioconjug. Chem..

[23] Turner K.B., Hagan N.A., Kohlway A.S., Fabris D. (2006). Mapping noncovalent ligand binding to stemloop domains of the HIV-1 packaging signal by tandem mass spectrometry. J. Am. Soc. Mass Spectrom..

