# Supplementary material for: In Vitro Evaluation of Bis-3-Chloropiperidines as RNA Modulators Targeting TAR and TAR-Protein Interaction
Source: Int J Mol Sci. 2022 Jan 6;23(2):582. doi: 10.3390/ijms23020582 (PMC8776071; doi:10.3390/ijms23020582)
Supplement: Supplementary file 1 [file ijms-23-00582-s001.zip › ijms-1528759-supplementary.pdf]

Supporting Information

In vitro evaluation of bis-3-chloropiperidines as RNA modulators targeting TAR and TAR-protein interaction

Alice Sosic [1], Giulia Olivato [1], Caterina Carraro [1], Richard Göttlich [2], Dan Fabris [3], Barbara Gatto [1*]

[1] Department of Pharmaceutical and Pharmacological Sciences, University of Padova, Via Francesco Marzolo 5, 35131 Padova (Italy); caterina.carraro.2@phd.unipd.it (C.C.), barbara.gatto@unipd.it (B.G.)
[2] Institute of Organic Chemistry, Justus Liebig University Giessen, Heinrich-Buff-Ring 17, 35392 Giessen (Germany); Richard.Goettlich@org.chemie.uni-giess (R.G.)
[3] Department of Chemistry, University of Connecticut, 55 North Eagleville Rd., Storrs, CT 06269 (USA); dan.fabris@uconn.edu (D.F.)
* Correspondence: barbara.gatto@unipd.it

Table of content

Figure S1 p.2

Table S1 p.3

Figure S2 p.4

Figure S1. Representative ESI-MS spectrum obtained from mixtures of equimolar concentrations of TAR RNA and NC protein.

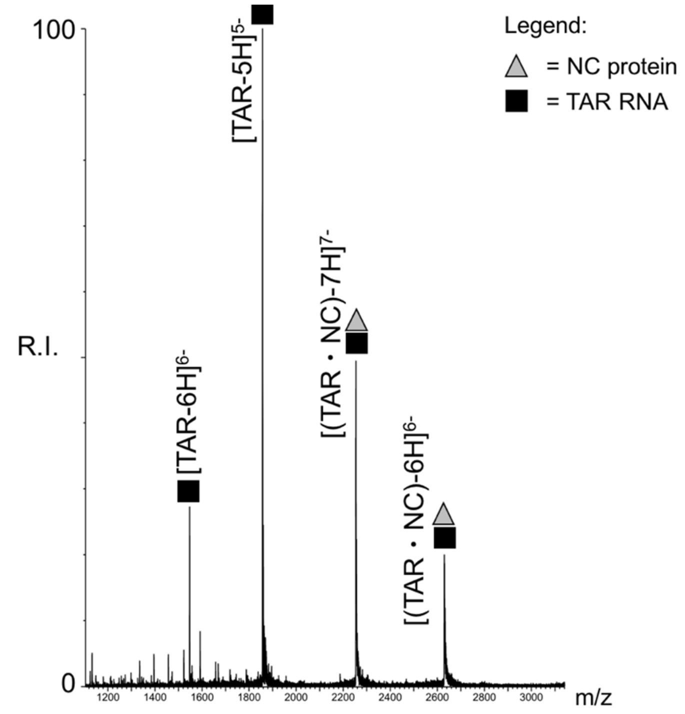

In addition to free RNA, the formation of the non-covalent 1:1 TAR•NC complex was readily detected with a mass of 15786.8 Da, which matched very closely the average mass value of 15786.8 Da calculated from the RNA and protein sequences including two Zn(II) ions.

Table S1. Digestion products obtained by treating B-CeP 2- or B-CeP 3-reacted TAR RNA with RNAse A corresponding to bridged RNA fragments.

Label	Compound	Bridged RNA fragments	Symbol	Exp. mass (u)	Calc. mass (u)
**XL1**	B-CeP **2**	G10:C13 + **2**$_{B}$ + G16:C21	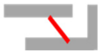	3664.77	3664.77
	B-CeP **3**	G10:C13 + **3**$_{B}$ + G16:C21		3678.79	3678.79
**XL2**	B-CeP **2**	G1:C3 + **2**$_{B}$ + U26:C28	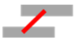	2277.56	2277.56

The Table summarizes the bi-functional alkylation products bridging base-paired regions within TAR RNA hairpin detected by treating TAR RNA with either B-CeP **2** or **3**, followed by RNAse A digestion. Product labels refer to Figure 4 in the main text. Oligonucleotides products are indicated by the first and last base, separated by colon. Product labels are also reported on the hairpin secondary structure cartoon in Figure 4C.

Figure S2. Schematic representation of the cleavage sites detected upon RNAse A digestion within TAR secondary structure.

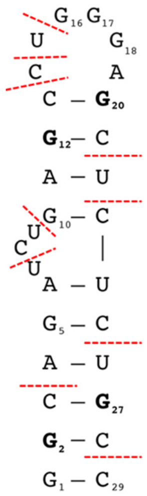

According to RNAse A specificity, which hydrolyzes preferentially single-stranded C and U, the detected fragments corresponded to stretches of TAR cleaved only after pyrimidine residues. The cartoon illustrates the location of the cleavage sites (dashed lines) detected in the spectra shown in Figure 4A and B.
